# Retrograde esophageal endoscopic submucosal dissection via gastrostomy without dilation using a thin therapeutic endoscope

**DOI:** 10.1055/a-2885-8164

**Published:** 2026-06-12

**Authors:** Yuichiro Mikuriya, Kazuo Shiotsuki, Nobuhisa Minakata, Kohei Takizawa, Shin Maeda

**Affiliations:** 1 Department of Gastroenterology 91321 Kanagawa Cancer CenterYokohamaKanagawaJapan; 2 Department of Gastroenterology 26438 Yokohama City University School of Medicine Graduate School of MedicineYokohamaKanagawaJapan

## E-Videos

Retrograde esophageal endoscopic submucosal dissection via gastrostomy without dilation using a thin therapeutic endoscope.

## A case description



**Video 1** Use of a thin therapeutic endoscope for retrograde transgastrostomy esophageal endoscopic submucosal dissection without tract dilation.


**Fig. 1:**
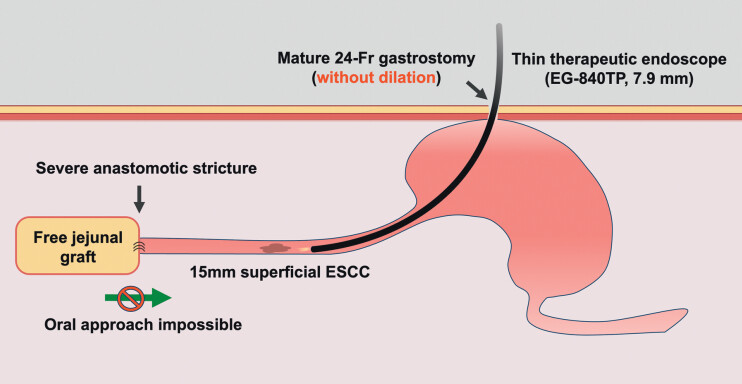
Schematic illustration of a retrograde transgastrostomy approach for esophageal endoscopic submucosal dissection using a thin therapeutic endoscope.

Endoscopic treatment of superficial esophageal squamous cell carcinoma distal to strictures is challenging when oral access is impossible. 1​ Although several techniques have been reported, most require adjunctive procedures, such as balloon dilation or gastrostomy tract enlargement, which may increase the risk of perforation and peristomal complications. 2​ 3​ ​4​ A thin therapeutic endoscope (EG-840TP; Fujifilm, Tokyo, Japan; outer diameter, 7.9 mm) enables esophageal ESD adjacent to strictures. 5​ Here, we report the use of this endoscope for retrograde ESD through a mature gastrostomy without tract dilation, as a less invasive alternative ( Fig. 1 and Video 1 ).

**Fig. 2:**
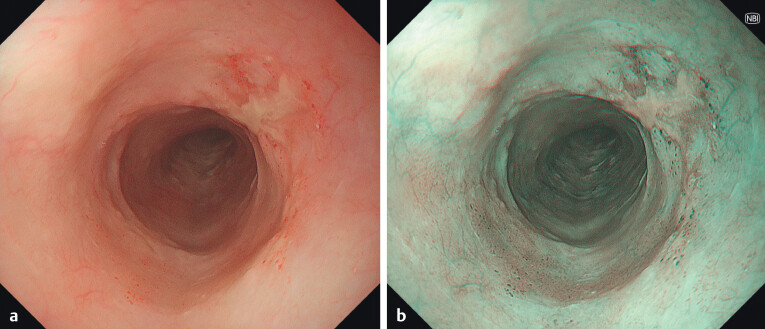
( **a** ) White-light endoscopy showing a 15-mm superficial lesion distal to the anastomotic stricture. ( **b** ) Narrow-band imaging of the lesion suggesting superficial squamous cell carcinoma.

A 55-year-old woman with cervical esophageal squamous cell carcinoma underwent 24-Fr percutaneous endoscopic gastrostomy (PEG) using a bumper-type catheter (Ideal Button, Olympus, Tokyo, Japan) before chemoradiotherapy. Due to the persistent residual tumor, salvage pharyngolaryngoesophagectomy with free jejunal reconstruction was performed. Subsequently, a severe anastomotic stricture developed, through which only an ultrathin transnasal endoscope could pass. Surveillance endoscopy revealed a 15-mm superficial esophageal squamous cell carcinoma distal to the stricture ( Fig. 2 ).

**Fig. 3:**
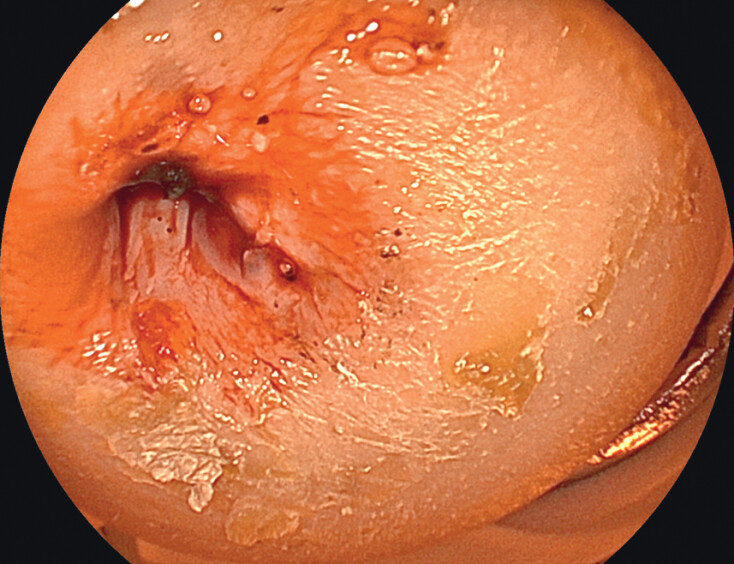
The mature 24-Fr gastrostomy tract after PEG tube removal.

Antegrade oral ESD was not feasible because even the EG-840TP endoscope could not pass through the stricture, and balloon dilation was considered high risk. Therefore, a retrograde ESD via gastrostomy was planned. After removing the PEG tube, the EG-840TP endoscope was easily passed through the gastrostomy without tract dilation and advanced retrogradely into the lesion ( Fig. 3 ). ESD was performed using standard techniques. En bloc resection was completed within 23 minutes without complications. Pathological findings revealed squamous cell carcinoma invading the muscularis mucosae, with negative horizontal and vertical margins and no lymphovascular invasion. A new 24-Fr PEG tube was placed to allow early enteral feeding.

This case suggests that retrograde ESD through a gastrostomy using a thin therapeutic endoscope is safe and feasible without dilation of either the stricture or the tract when oral access is impossible.

Endoscopy_UCTN_Code_TTT_1AO_2AG_3AD.
